# Short Segments of Electrospun Nanofibers Loaded with Curcumin Can Protect the Cells in Spheroids against Oxidative Stress

**DOI:** 10.1021/acsanm.6c00093

**Published:** 2026-02-24

**Authors:** Yuxuan Meng, Min Hao, Younan Xia

**Affiliations:** † School of Chemistry and Biochemistry, 1372 Georgia Institute of Technology, Atlanta, Georgia 30332, United States; ‡ The Wallace H. Coulter Department of Biomedical Engineering, Georgia Institute of Technology and Emory University, Atlanta, Georgia 30332, United States; § Department of Materials Science and Engineering, 1466 Johns Hopkins University, Baltimore, Maryland 21218, United States; ∥ Department of Biomedical Engineering, Johns Hopkins University, Baltimore, Maryland 21218, United States

**Keywords:** oxidative stress, curcumin delivery, cellular protection, electrospun fibers, antioxidant therapy

## Abstract

Oxidative stress in damaged or inflamed tissues presents a major barrier to the efficacy of cell-based therapies by impairing cell viability, function, and engraftment. Herein, we demonstrate a nanofiber-integrated three-dimensional spheroid platform that delivers Curcumin (Cur), a natural antioxidant, for cellular protection. Cur can be encapsulated in electrospun polycaprolactone (PCL) fibers, which are processed into short segments and coassembled with human mesenchymal stem cells to form spheroids. The integrated fibers enable a two-phase release profile of Cur while preserving spheroid morphology and maintaining cell organization. Under H_2_O_2_-induced oxidative stress, Cur-PCL-integrated spheroids showed improved cell viability and reduced mitochondrial reactive oxygen species compared with untreated controls. Unlike conventional nanoparticle-based systems that often rely on inefficient cellular uptake and can suffer from limited penetration in 3D aggregates, this fiber-segment approach provides a physically retained intraspheroidal depot that enables localized cytoprotection while preserving spheroid integrity, offering a scalable and injectable strategy for engineering resilient cell constructs. The system holds promise for improving the therapeutic performance of stem cell therapies in oxidative microenvironments associated with tissue injury and regeneration.

## Graphical abstract

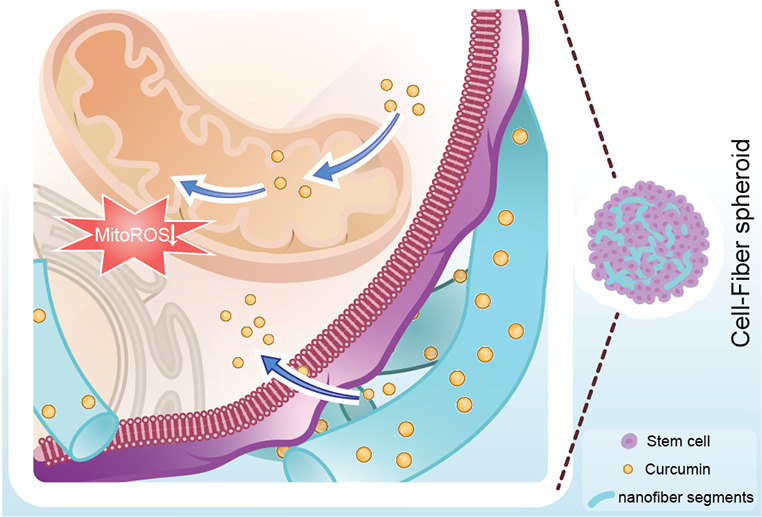

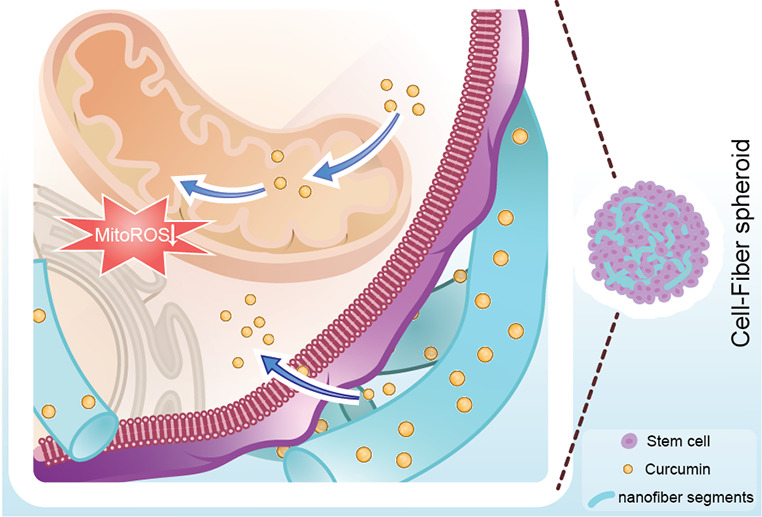

## 1 Introduction

Three-dimensional (3D) cell spheroids have emerged as a powerful tool in tissue engineering, regenerative medicine, and cell-based therapies due to their ability to recapitulate the architecture and microenvironment of native tissues more closely than conventional two-dimensional (2D) cultures.^1,2^ By facilitating physiologically relevant cell–cell and cell-extracellular matrix (ECM) interactions, spheroidal architecture can improve cellular viability, retention of stemness, and differentiation potential.^3,4^ When used for transplantation, spheroid-based delivery of cells has shown promise in enhancing cell engraftment, paracrine signaling, and immunomodulatory effects, making them a clinically attractive vehicle for cell-based therapies.^5–8^ Despite these advantages, the therapeutic efficacy of spheroids remains to be limited by the oxidative stress in pathological tissues such as ischemic, inflamed, or wounded sites.^9,10^ Typically, elevated levels of reactive oxygen species (ROS), including superoxide, hydrogen peroxide (H_2_O_2_), and hydroxyl radical, can result in mitochondrial dysfunction, DNA damage, and ultimately apoptosis in the transplanted cells.^11,12^ While spheroidal organization provides some physical buffering, it is inadequate to protect the cells from persistent oxidative challenge, underscoring the need for strategies capable of endowing spheroids with intrinsic antioxidant capacity.^13^

Antioxidant strategies span small-molecule antioxidants (e.g., polyphenols and phenolic compounds), enzymatic or enzyme-mimetic systems (e.g., superoxide dismutase/catalase-inspired approaches), and redox-active biomacromolecules that preserve intracellular redox buffering capacity.^14–16^ Recent work has also highlighted the promise of biomass-derived, redox-active materials in biomedical settings.^17–19^ For example, antioxidative lignin-based materials have been reported to preserve glutathione and modulate redox-associated signaling pathways (e.g., IRS1/PI3K/AKT) in disease-relevant models, underscoring the broader utility of antioxidant material design for mitigating oxidative stress-related damage.^20^

Among various antioxidant agents, curcumin (Cur), a polyphenolic compound extracted from *Curcuma longa*, stands out for its potent ROS-scavenging activity, along with anti-inflammatory and cytoprotective properties.^21,22^ Cur can scavenge free radicals, chelate metal ions, and modulate redox-sensitive signaling pathways such as Nrf2 and PI3K/Akt.^23–25^ However, the use of Cur is compromised by its poor solubility, low bioavailability, and instability under physiological conditions, greatly limiting its therapeutic application.^26,27^ Encapsulation in micro/nanoparticles has been widely explored to address these shortcomings, yet such systems rely on cellular internalization to deliver the cargo, a process that is inefficient, cell-type dependent, and sometimes detrimental to cellular function.^28,29^ Moreover, micro/nanoparticle-mediated delivery primarily targets intracellular ROS, whereas extracellular or membrane-associated ROS frequently constitute the first line of oxidative stress encountered by the transplanted cells.^30–32^ Electrospun polymer nanofibers offer an attractive alternative for Cur delivery, owing to their high specific surface areas, compatibility with hydrophobic drugs, and sustained release properties.^33^ Beyond serving as a drug reservoir, fragmenting electrospun mats into short, high-aspect ratio segments enables functions that spherical micro/nanoparticles cannot readily provide once integrated into 3D cell aggregates. Unlike conventional approaches that employ fibers as external scaffolds, embedding drug-loaded fiber segments within spheroids ensures direct integration of therapeutic carriers within the 3D architecture. The high-aspect ratio segments can physically bridge cells during aggregation/compaction, reinforcing spheroid cohesion, while their ECM-like fibrillar morphology promotes close cell-material contact. Their random arrangement can also create porous transport pathways that alleviate diffusion limitations in the spheroid interior. Finally, distributing the segments throughout the spheroid establishes an intraspheroidal depot for spatially localized, sustained Cur release, supporting improved cellular outcomes under oxidative stress.

Herein, we demonstrate the rational fabrication of a fiber-integrated spheroid system in which Cur-loaded polycaprolactone (PCL) nanofibers are fragmented into short segments and then coassembled with human mesenchymal stem cells (MSCs) to form compact, uniform spheroids (Scheme 1). This intraspheroidal fiber-segment depot enables localized Cur release and provides antioxidant protection under oxidative stress, as reflected by reduced mitochondrial ROS and improved cell viability compared with controls. Meanwhile, the physical presence of the fiber segments offers structural support and close cell-material contact within the spheroid microenvironment. Together, these results support a simple strategy to enhance the robustness of therapeutic cell spheroids in regenerative medicine.

**Scheme 1:**
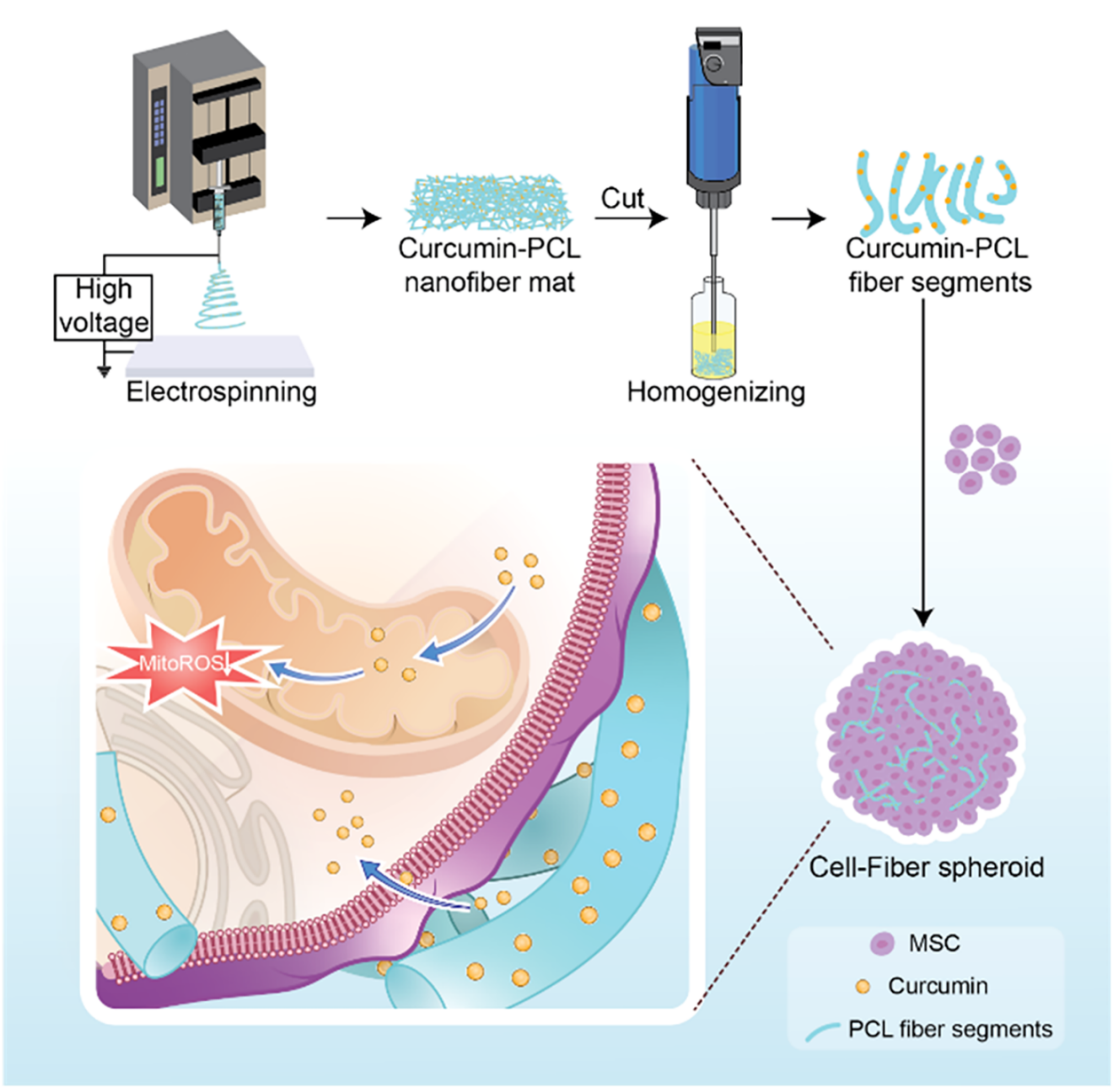
Schematic Illustrating the Fabrication of Cell Spheroids Mixed with Cur-Loaded Segments of Electrospun PCL Nanofibers for Cellular Protection

## 2 Experimental Section

### 2.1 Chemicals and Materials

PCL (M_w_ ≈ 80,000), dichloromethane (DCM), *N*, *N*-Dimethylformamide (DMF), rhodamine B, Cur, ethylenediamine (EDA), potassium persulfate and H_2_O_2_ were all obtained from Sigma-Aldrich and used as received. Phosphate-buffered saline (PBS), collagenase, ethylbenzthiazoline-6-sulfonate (ABTS), 1,1-diphenyl-2-picrylhydrazyl free radical (DPPH), Minimum Essential Medium α (α-MEM), fetal bovine serum (FBS), penicillin-streptomycin, calcein AM, ethidium homodimer-1 (EthD-1), paraformaldehyde, 4′,6-diamidino-2-phenylindole (DAPI), Phalloidin-iFluor 488, MitoSOX mitochondrial superoxide indicator and Hoechst 33342 were purchased from Thermo Fisher Scientific. Cell counting kit 8 (CCK8) was obtained from Dojindo. Isopropyl alcohol (IPA) was purchased from VWR. All aqueous solutions were prepared with deionized (DI) water with a resistivity of 18.2 MΩ·cm at room temperature.

### 2.2 Preparation of PCL Fiber Segments Loaded with Cur

Cur-loaded PCL fiber segments were fabricated through a combination of electrospinning and homogenization. For electrospinning, a PCL solution at 10 wt % was prepared by dissolving PCL in a 9:1 (v/v) mixture of DCM and DMF under magnetic stirring for 12 h at room temperature. Subsequently, Cur was introduced into the PCL solution at a percent of 20 wt % relative to the polymer mass, followed by additional stirring for 12 h to ensure homogeneity. The resulting PCL-Cur solution was electrospun using a standard electrospinning setup, with a 22G blunt needle and a feeding rate of 0.6 mL h^–1^ under an applied voltage of 15 kV. Fibers were collected on a flat aluminum foil for 3 h, and the resultant mats were peeled off and placed in a vacuum oven overnight at 25 °C to remove residual solvent. To improve the surface hydrophilicity, the mats were treated using a plasma cleaner (Plasma Etch PE50, Carson City, NV) for 2 min on each side. For homogenization, the mats were first cut into small pieces and treated with EDA working solution (10% v/v in IPA) under vigorous shaking at 150 rpm for 24 h. The treated mats were then homogenized using an ultrasonic homogenizer (Qsonica Q125 Sonicator, Qsonica LLC, Newtown, CT) equipped with a 20 kHz probe and a 1/8 in. tapered microtip probe. The homogenization was performed under ice bath cooling for 10 min, using on/off cycles of 10/5 s at 100% amplitude. The resulting suspension was then centrifuged at 11000 rpm to remove residual EDA and the collected fiber segments were resuspended in 2 mL PBS. For fluorescence visualization, a portion of the obtained Cur-loaded PCL fiber segments was incubated in a rhodamine B solution (100 μg/mL) for 12 h at 25 °C (protected from light) to absorb this dye. The labeled segments were then washed five times with PBS to remove unbound dye prior to imaging.

### 2.3 Cur Release Kinetics

The release profile of Cur from the Cur-loaded fiber segments was assessed under physiological and enzymatic conditions. Briefly, 100 μg of the Cur-loaded fiber segments were suspended in either 1 mL of PBS or collagenase solution (50 μg/mL) and incubated at 37 °C. The release medium was collected at predetermined time intervals for analysis, and an equal volume of fresh PBS or collagenase solution was added to maintain a constant volume and consistent release conditions throughout the study. The concentration of the released Cur was quantified by measuring absorbance at 425 nm using a multimode plate reader (BioTek Synergy H1, Agilent Technologies). Release studies were performed with *n* = 3 independent samples.

### 2.4 Free Radical Scavenging Assay

For the ABTS assay, the free radical was generated by incubating 12 mg of ABTS and 4 mg of potassium persulfate in 2 mL of water for 12 h in the dark. Afterward, a mixture containing 15 μg of Cur-loaded PCL fiber segments, 5 μL of ABTS solution with free radicals, and 985 μL of DI water was incubated in the dark for 15 min. For the DPPH assay, 15 μg of Cur-loaded PCL fiber segments were incubated with 1 mL DPPH (0.1 mM) in the dark for 30 min. Following incubation, to minimize matrix-related optical artifacts from suspended segments (e.g., scattering/turbidity), the mixtures were centrifuged to pellet and remove all fiber segments. The absorbances of the above samples were then measured using a ultraviolet–visible **(**UV–vis) spectrometer.

### 2.5 Cell Culture and Assembly of Cell-Fiber Spheroids

MSCs were ordered from a commercial company (Lonza, Basel, Switzerland) and were recovered from cryopreservation. Typically, MSCs were cultured in α-MEM supplemented with 10% FBS and 1% penicillin/streptomycin under standard culture conditions (37 °C, 5% CO_2_). An AggreWell400 24-well plate (STEMCELL Technologies, Canada) was used for the fabrication of the Cell-Fiber spheroids following the manufacturer’s protocol. Briefly, 15 μg of the Cur-loaded PCL fiber segments were added to 2 mL of growth medium with 10^6^ MSCs. The mixture was transferred into one well of the AggreWell400 plate. The plate was pre-treated with an anti-adherent solution (STEMCELL Technologies, Canada) at 37 °C for 2 h. Following seeding, the plate was centrifuged at 1500 rpm for 5 min and then incubated under standard culture conditions. Cell-fiber spheroids were formed within 24 h, harvested by gentle pipetting, and transferred to centrifuge tubes for subsequent staining experiments.

### 2.6 Immunofluorescence Staining

The spheroids were collected by centrifugation (1500 rpm, 5 min) and washed three times with PBS. The spheroids were then fixed with 4% paraformaldehyde for 10 min, followed by permeabilization with 0.25% Triton X-100 for 5 min. Subsequently, the spheroids were incubated with Phalloidin-iFluor 488 (1:100 dilution) for 30 min and then washed three times with PBS. Afterward, DAPI was used to stain cell nuclei for 10 min. The stained spheroids were visualized using a laser scanning confocal microscope for fluorescence imaging.

### 2.7 Cell Live/dead Staining

After 48 h of culture, the Cell-Fiber spheroids were collected in centrifuge tubes and washed three times with PBS. The spheroids were then incubated in a serum-free α-MEM medium containing 2 μM calcein AM and 1 μM EthD-1 at 37 °C for 20 min. The resultant spheroids were washed with PBS and analyzed using a laser scanning confocal microscope.

### 2.8 CCK8 Assay

After 48 h of culture, the culture medium was replaced with a serum-free α-MEM medium containing 10% (v/v) CCK8 solution. The spheroids were then incubated at 37 °C for 1 h. Following incubation, the absorbance of the medium was measured at 450 nm using a multimode plate reader (BioTek Synergy H1, Agilent Technologies) to assess cell viability. For each group, *n* = 5 wells were analyzed.

### 2.9 Simulation of Oxidative Stress Conditions

To simulate oxidative stress, cell-fiber spheroids were cultured for 24 h and then treated with α-MEM supplemented with 10% FBS, 1% penicillin/streptomycin, and 500 nM H_2_O_2_ (diluted 1:1000) for another 24 h. Cell viability following H_2_O_2_ treatment was assessed using the CCK8 assay, as described above.

### 2.10 Mitochondrial Superoxide Live Cell Tracking

The spheroids were incubated with α-MEM medium containing MitoSOX mitochondrial superoxide indicator (1:500 dilution) for 20 min. Subsequently, the spheroids were stained with Hoechst 33342 (1:1000 dilution) for 10 min to visualize cell nuclei. After that, the spheroids were evaluated using a laser scanning confocal microscope. Quantification was performed on *n* = 5 spheroids per group.

### 2.11 Characterizations

The morphologies of the electrospun mats and fiber segments were analyzed using scanning electron microscopy (SEM, Hitachi SU8230, Japan). All samples were coated with a Hummer 6 Au/Pd sputter (Anatech, Union City, CA) before imaging. The Fourier-transform infrared (FTIR) spectra were obtained using a Shimadzu IRAffinity-1 spectrometer (Shimadzu, Kyoto, Japan). The UV–vis spectra were recorded on a Cary 60 UV–vis spectrometer (Agilent Technologies). The fluorescence micrographs were acquired using a laser scanning confocal microscope (Zeiss LSM 900, Zeiss, Oberkochen, Germany).

### 2.12 Statistical Analysis

All the results are presented as mean ± standard deviations unless otherwise stated. The number of independent samples (n) for each experiment is provided in the corresponding Experimental subsections and/or figure plots. Statistical analysis was performed using one-way ANOVA followed by Tukey’s comparison test, using GraphPad Prism 10. A p-value less than 0.05 (*p* < 0.05) was considered statistically significant. Significance is indicated in the figures as follows: ns, not significant; *p* < 0.05 (*); *p* < 0.01 (**); *p* < 0.001 (***); *p* < 0.0001 (****).

## 3 Results and Discussion

### 3.1 Fabrication and Characterizations of the Cur-Loaded PCL Fiber Segments

The short segments of Cur-PCL nanofibers were fabricated by a combination of electrospinning and homogenization. Initially, nonwoven mats of Cur-PCL fibers were produced by mixing Cur with PCL in a 9:1 (v/v) mixture of DCM and DMF, followed by electrospinning with the traditional setup. Cur and PCL were co-dissolved and electrospun from a homogeneous solution, which is expected to yield a largely matrix-dispersed Curdistribution upon rapid jet solidification.^34,35^ As shown by the SEM images in Figures 1A and S1, the fibers with and without Cur exhibited similar morphologies, with a diameter of 851.8 ± 157.4 nm, indicating that the incorporation of Cur at a level of 20 wt % did not compromise the electrospinning process. Upon homogenization, the Cur-PCL fibers were fragmented into short segments with an average length of 12.19 ± 5.95 μm (Figures 1B and S2A). To further characterize the morphological stability of the Cur-PCL fibers post-homogenization, we quantified the fiber segment diameters. As shown in Figure S2B, the average diameter of the homogenized fiber segments was 814.9  ±  216.8 nm, comparable to that of the as-spun fibers, indicating that homogenization predominantly truncates fiber length while preserving fiber thickness and overall morphology. Additionally, the homogenized Cur-PCL segments exhibit an average aspect ratio of ca. 15, a range that supports physical retention within spheroids during aggregation/compaction without disrupting spheroid formation.^36^ Additional SEM images (Figure S3) were also provided to present representative fields with higher segment density and morphology diversity.

**Figure 1:**
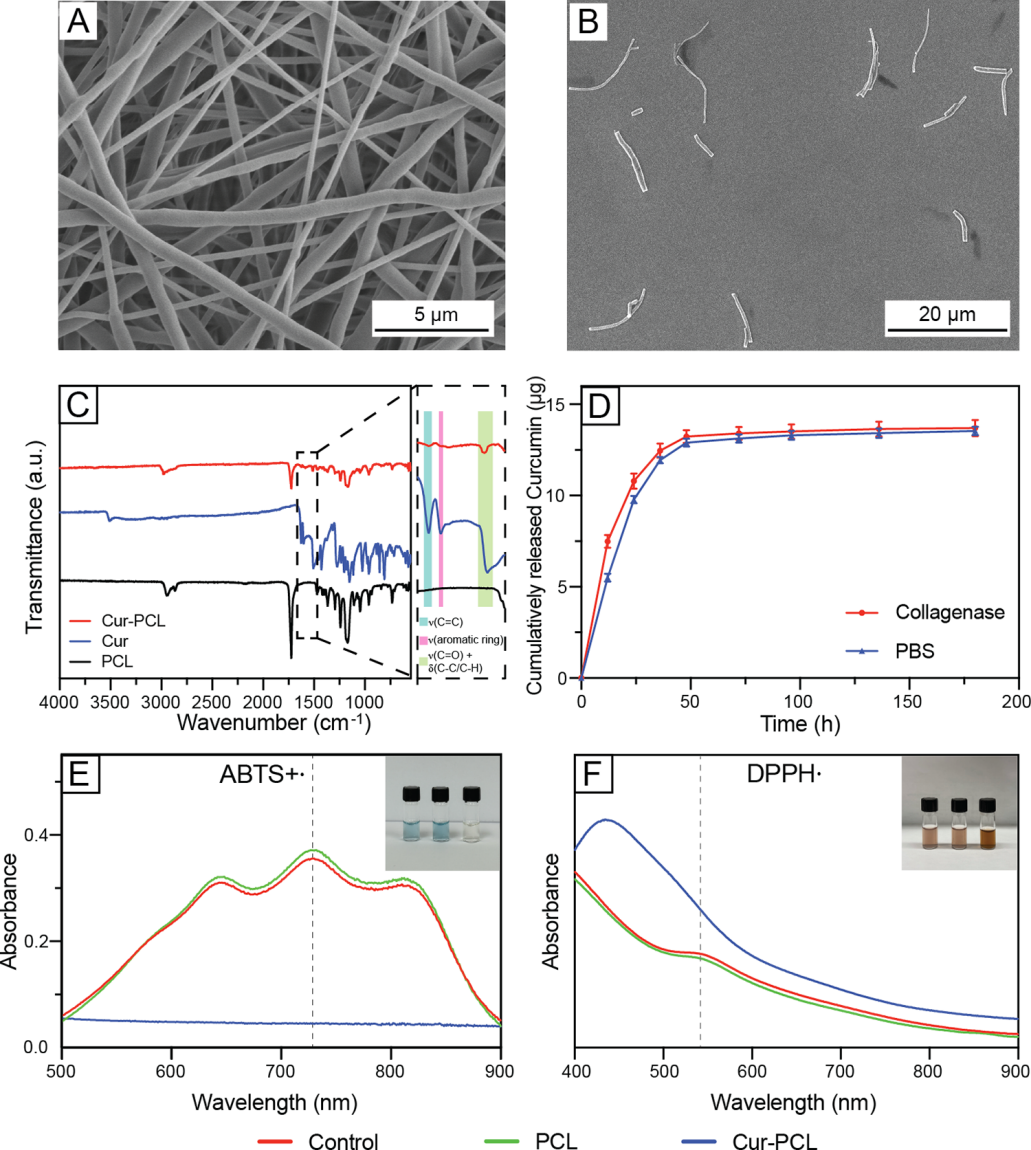
SEM images of (A) the random electrospun Cur-PCL nanofiber mat prepared from a 20 wt % Cur-loaded PCL solution in DCM/DMF (9:1 v/v) and (B) fiber segments fabricated using homogenizer. (C) FTIR spectra of PCL electrospun fiber mats (black), Cur powder (blue), and Cur-PCL fiber mats (red). Characteristic peaks are highlighted: C=C stretching at 1625 cm^–1^ (blue region), aromatic ring vibrations at 1598 cm^–1^ (pink region), and C=O stretching and in-plane deformations of the C–C and C–H bonds within the keto–enol moiety at 1510 cm^–1^ (green region). (D) Cumulative release of Cur from the Cur-PCL fiber segments in PBS solution (blue) and collagenase solution (red). (E, F) UV–vis spectra of the radicals incubated with the control group (radicals only), PCL fiber segments, and Cur-PCL fiber segments. The insets show photographs of the control, PCL, and Cur-PCL samples arranged from left to right.

As shown by the FTIR spectrum in Figure 1C, the Cur-PCL fibers showed a strong C=O stretching band at 1720 cm^–1^, corresponding to the characteristic peak of PCL.^37^ Additionally, in the magnified region of Figure 1C, Cur-specific peaks, including C=C stretching (1625 cm^–1^), aromatic ring vibration (1598 cm^–1^), and mixed vibrations involving C=O stretching and in-plane deformations of the C–C and C–H bonds within the keto–enol moiety (1510 cm^–1^),^38,39^ also appeared in the spectrum of Cur-PCL fibers but were absent from pure PCL fibers. These data confirmed that Cur was successfully incorporated during the electrospinning process.

The encapsulation efficiency (EE%) of Cur within the fibers was measured using UV–vis spectroscopy. After dissolution of the Cur-PCL fiber segments in chloroform, the Cur content was quantified by comparing the measured absorbance with the standard curve in Figure S4. The EE% of Cur was determined to be 58.6 ± 7.6%, indicating that a large proportion of Cur could be incorporated into the PCL fibers without compromising the electrospinning process.

The *in vitro* release profile of Cur from the Cur-PCL fiber segments was measured under both physiological (PBS) and enzymatic (collagenase) conditions, and the results are plotted in Figure 1D. An initial burst release was observed within the first 24 h, reaching ca. 50% of the encapsulated content under both conditions. This release is primarily caused by the rapid diffusion of Cur molecules loosely associated with or near the fiber surface.^40,41^ After this phase, a gradual and sustained release occurred, which can be attributed to the continued diffusion of Cur through the hydrophobic PCL matrix.^40,41^ The release behavior of Cur is governed by physical encapsulation and drug-polymer affinity within the hydrophobic PCL matrix. As a hydrophobic molecule, Cur exhibits a favorable affinity for PCL, promoting its retention within the hydrophobic fiber interior. These noncovalent hydrophobic and van der Waals interactions help reduce rapid leaching, contributing to the observed biphasic release profile: an initial burst phase attributed to surface-associated Cur, followed by a sustained diffusion-controlled release from the fiber core.^40,42^ In the presence of collagenase, a slightly higher level of release was observed relative to the case of PBS, which can be related to partial degradation of the PCL matrix. This trend suggests that Cur release can be regulated, especially in environments with elevated enzymatic activity, such as inflamed tissues. Taken together, these results demonstrate that Cur is gradually released from the PCL fiber segments, providing both an initial burst and a sustained supply that are advantageous for therapeutic applications. This release window is best suited for cytoprotection during spheroid formation/handling and the early post-transplant/early engraftment period, when transplanted cells are often most vulnerable to acute oxidative stress and inflammatory injury, whereas longer-term *in vivo* therapies would require further carrier tuning to extend release duration.

As previously mentioned, ROS primarily encompasses superoxide, H_2_O_2_, and hydroxyl radical. Cur, with phenolic hydroxyl groups and keto–enol moiety, can donate hydrogen atoms or electrons, thereby scavenging ROS and other free radicals.^43^ To this end, we evaluated the antioxidant activity of the Cur-PCL fiber segments using well-known free radical reactions (Figures 1E,F, and S5). Specifically, the cationic radical ABTS+· was generated and confirmed by its absorption peak at 734 nm.^44^ Upon addition of Cur-PCL fiber segments, the intense blue color of the ABTS+· solution gradually faded away, and the corresponding absorbance peak eventually diminished, indicating that the Cur-PCL fiber segments were able to donate electrons to neutralize the radical. Similarly, another stable free radical, DPPH· with a characteristic peak at 517 nm was used to the test.^11^ Again, the introduction of Cur-PCL fiber segments resulted in the eventual disappearance of the characteristic peak. These results demonstrated that the Cur-PCL fiber segments retain the antioxidant functionality of Cur, protecting cells from oxidative stress.

### 3.2 Fabrication of Cell-Fiber Spheroids

The assembly of the cell-fiber spheroids was conducted by following the procedure outlined in Figure 2A. Briefly, MSCs were digested into a suspension of single cells and collected from the culture flask. The Cur-PCL fiber segments were then mixed thoroughly mixed with the MSCs to achieve a uniform distribution and close contact between the fibers and cells. The mixture was then transferred into an Aggrewell plate, where microwells facilitated controlled aggregation of the cells and fibers into spheroids. Specifically, spheroid assembly in Aggrewell microwells is initiated by centrifugation-assisted loading, which concentrates cells and Cur-PCL fiber segments within confined microcavities and promotes rapid cell–cell contact. During incubation, cells undergo adhesion-mediated compaction and cytoskeletal reorganization to form a cohesive aggregate.^45^ The short fiber segments are incorporated primarily through physical colocalization in the microwells and remain retained by mechanical entrapment as the spheroid compacts. Compared with post-assembly surface adsorption approaches, this one-step co-assembly yields a mechanically cohesive microstructure with an intraspheroidal depot that both reinforces spheroid integrity and enables proximity-based Cur release.

**Figure 2:**
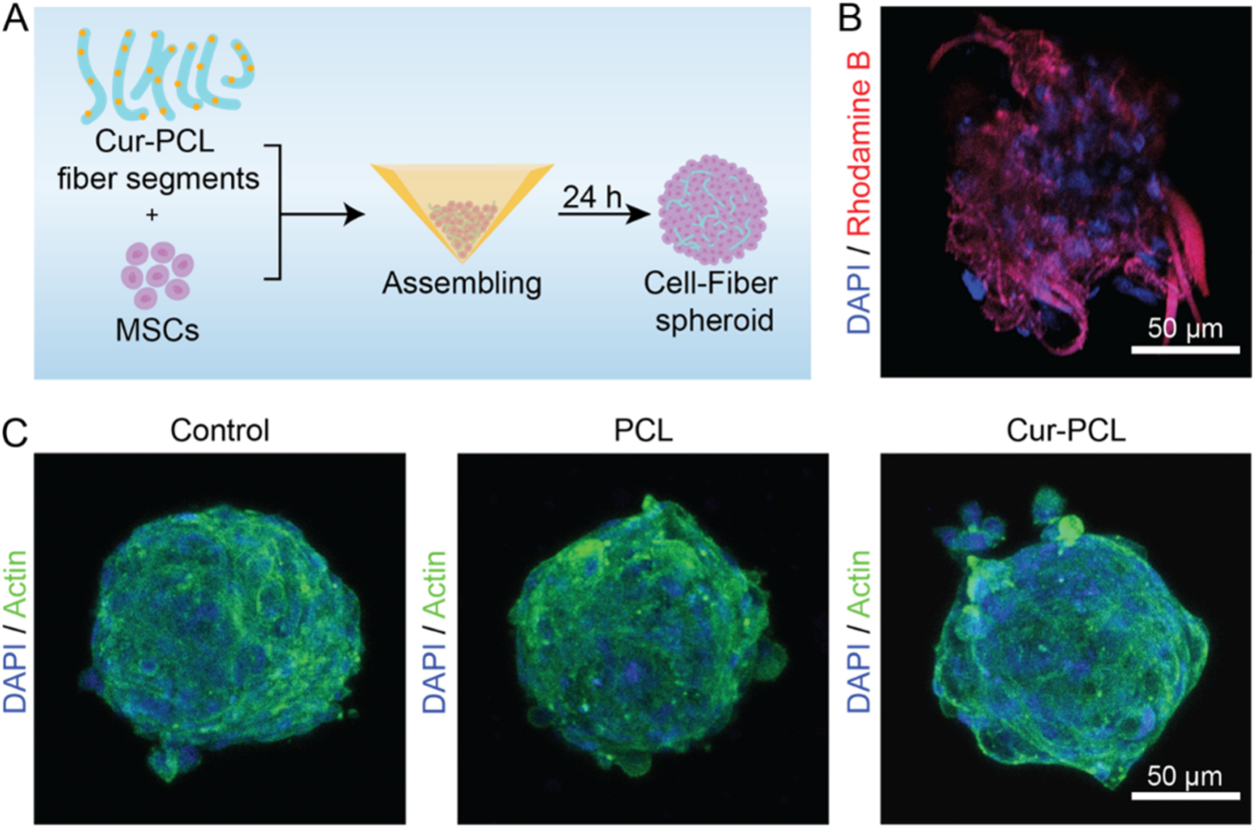
(A) Schematic illustration of the assembly process of Cur-PCL fiber segments and MSCs. (B) Immunofluorescent staining of DAPI (blue) in spheroids containing rhodamine-B labeled Cur-PCL fiber segments (red). (C) Immunofluorescent staining of Actin/DAPI in spheroids composed of MSCs only, PCL fiber segments plus MSCs, and Cur-PCL fiber segments plus MSCs.

After 24 h of incubation, cell-fiber spheroids were obtained. The successful incorporation and localization of fiber segments within the spheroids were confirmed by fluorescence imaging. To enable visualization, the Cur-PCL fiber segments were labeled by absorbing rhodamine B onto their surface, followed by repeated washing with PBS to remove unbound dye (Figure S6). As shown in Figure 2B, the rhodamine B-labeled Cur-PCL fiber segments were clearly distributed throughout the spheroids, demonstrating effective embedding and integration of fiber segments in the cellular aggregates. It should be noted that the fluorescence signal of rhodamine B in Figure 2B is used to qualitatively visualize fiber segment localization within 3D spheroids. The apparent feature thickness in confocal images can be larger than the SEM-measured fiber diameter due to optical limitations, including diffraction-limited lateral resolution, finite optical section thickness (z-integration), and scattering/out-of-focus signal in dense spheroids.

To evaluate the biological features of the cell-fiber spheroids, we established three groups: a control group comprised of MSC spheroids only, cell-fiber spheroids assembled from PCL fiber segments and MSCs (PCL), and cell-fiber spheroids assembled from Cur-PCL fiber segments and MSCs (Cur-PCL). Specifically, we cultured the spheroids for 24 h, followed by staining of the actins with phalloidin and nuclei with DAPI, respectively. As indicated by the confocal micrographs in Figure 2C, all three groups showed a well-defined, compact spheroidal structure with uniform morphology. Remarkably, there was no significant difference in overall shape or cellular organization for the spheroids among the three groups. The results indicated that the incorporation of either PCL or Cur-PCL fiber segments did not disrupt spheroid formation or alter cell morphology.

### 3.3 Biocompatibility of the Cur-PCL Fiber Segments

To evaluate the biocompatibility of the Cur-PCL fiber segments, we performed live/dead staining analysis of the cell-fiber spheroids after their formation (Figure 3A–C). Calcein AM was used to stain live cells in green while ethidium homodimer-1 was used to mark dead cells in red. The spheroids from all three groups showed a predominance of live cells with minimal cell death, demonstrating that the Cur-PCL fiber segments exhibit good biocompatibility and do not induce significant cytotoxicity.

**Figure 3:**
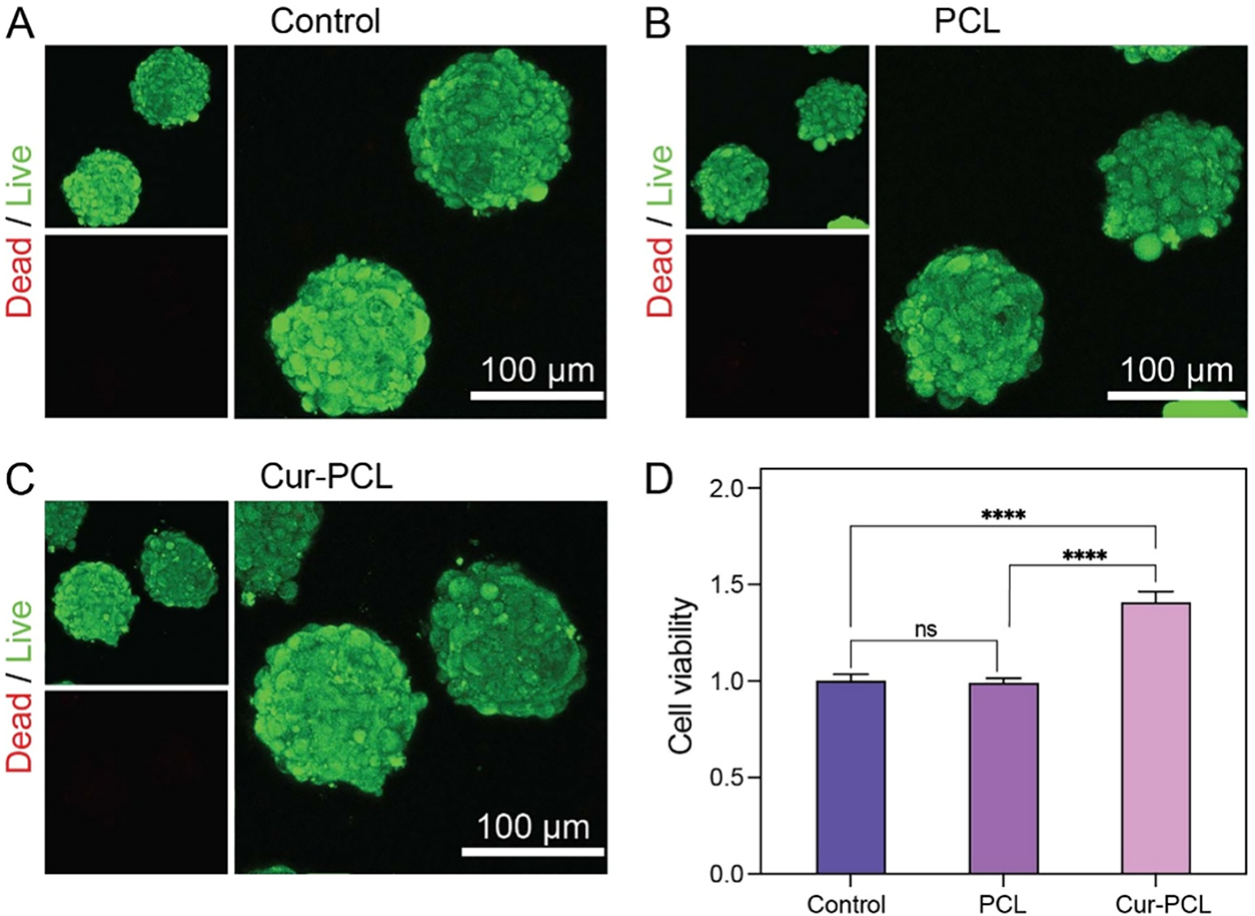
Live/dead staining of cells within the cell-fiber spheroids composed of (A) control (MSCs only), (B) PCL fiber segments plus MSCs, and (C) Cur-PCL fiber segments plus MSCs after 24 h of culture. (D) Quantitative analysis of cell viability using the CCK8 assay after 24 h of cell-fiber spheroids assembly.

Next, we evaluated the impact of the Cur-PCL fiber segments on cell proliferation using the CCK8 assay (Figure 3D). The results showed comparable cell viability between the control and PCL groups, while the Cur-PCL group exhibited a notable increase in cell proliferation. This enhancement might be attributed to the bioactivity of Cur, which has been reported to modulate cellular signaling pathways involved in survival and proliferation. This result further confirmed that the incorporation of Cur-PCL fiber segments into cell spheroids supports viability, establishing a basis for subsequent cell-based therapy.

### 3.4 Cellular Protection Against ROS as Mediated by the Cur-PCL Fiber Segments

Excessive ROS production, often present in injured or inflamed tissues, compromises cell survival and function in pathological environments.^46^ To evaluate whether sustained release of Cur from fiber segments could protect the cells under oxidative stress, we applied H_2_O_2_ to the cellular microenvironment to induce oxidative stress. After 24 h of H_2_O_2_ treatment, live/dead staining was performed to assess cell survival. The control group exhibited a high proportion of dead cells, indicating severe oxidative damage and loss of viability in response to H_2_O_2_ treatment (Figure 4A). In comparison, spheroids containing PCL fiber segments showed a slightly reduced red fluorescence signal (Figure 4B), suggesting that fiber incorporation did not exacerbate oxidative damage and may modestly support cellular organization. Notably, the most enhanced protective effect was observed in the Cur-PCL group (Figure 4C), where spheroids displayed a dominance of live cells with minimal cell death, demonstrating meaningful protection against ROS-induced apoptosis. A quantitative analysis of cell viability using the CCK8 assay further confirmed these observations. As shown in Figure 4D, spheroids containing Cur-PCL fiber segments exhibited significantly higher cell viability compared to both the PCL and control groups. Collectively, these results demonstrate that Cur-PCL fiber fragments provide effective antioxidant protection to MSC spheroids, thereby preserving cell viability under oxidative stress.

**Figure 4:**
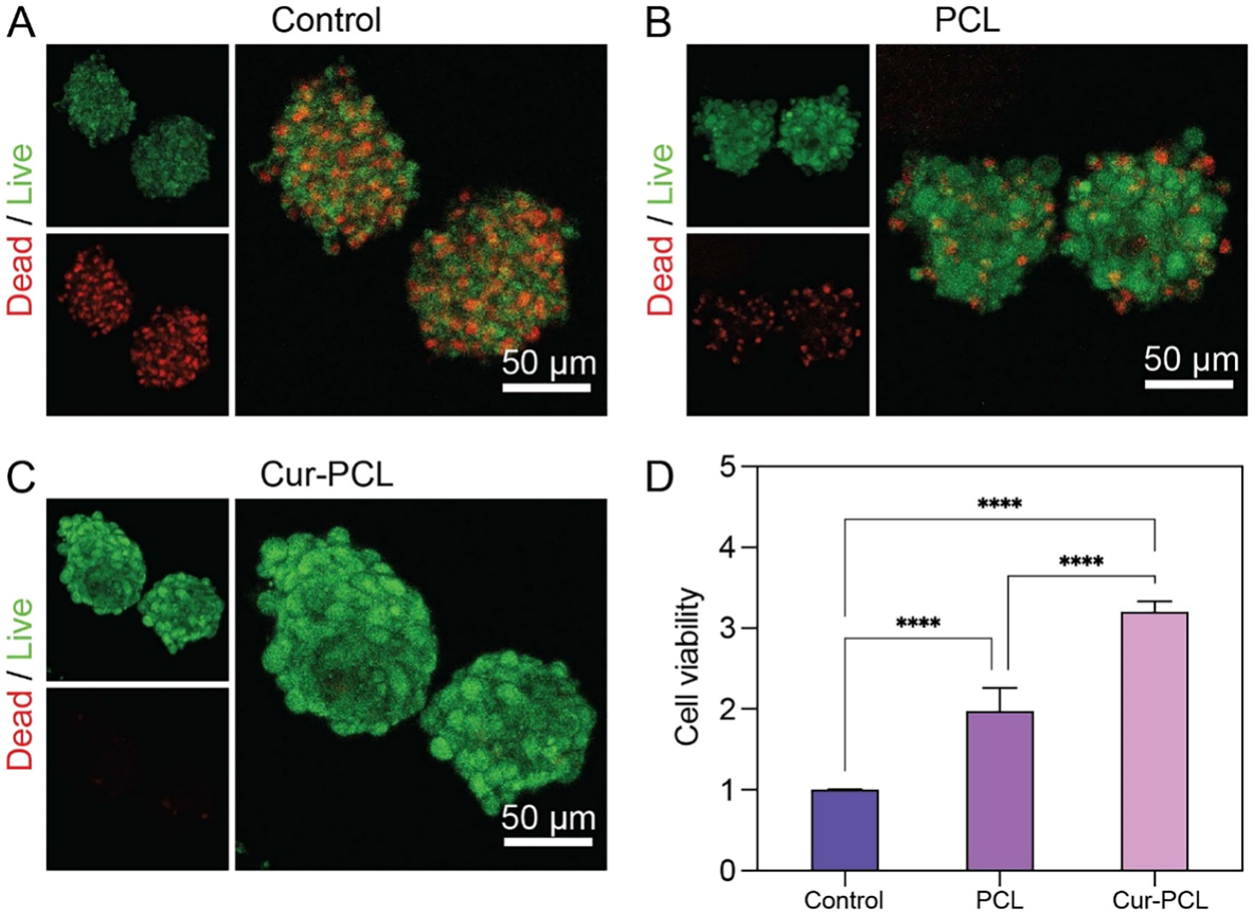
Live/dead staining of cell spheroids in the cases of (A) control (MSCs only), (B) PCL fiber segments, and (C) Cur-PCL fiber segments after co-culture with H_2_O_2_ solution. (D) Quantitative analysis of cell viability using the CCK8 assay after 24 h of H_2_O_2_ treatment.

In addition to assessing overall cell viability, we also evaluated mitochondrial-specific ROS levels to further investigate the protective effect of Cur-PCL fiber segments against oxidative stress. Mitochondria are a major source and target of ROS in cells in the case of elevated oxidative stress, where the mitochondrial homeostasis of a cell is prone to disruption, which then leads to apoptosis.^47,48^ As a selective fluorescent probe, mitochondrial superoxide (MitoROS) red was used to detect mitochondrial superoxide production. As shown in Figure 5A, the control group exhibited intense red fluorescence after 24 h of H_2_O_2_ exposure, indicating excessive ROS accumulation in response to the oxidative stress. Spheroids with the PCL fiber segments showed a reduction in the MitoROS level, suggesting that the PCL fibers provided a certain level of ECM-like structural support to help alleviate oxidative stress. In contrast, the Cur-PCL fiber segments dramatically reduced red fluorescence, highlighting their potent ability to suppress MitoROS formation, which could lead to apoptosis. Quantitative analysis (Figure 5B) of MitoSOX fluorescence intensity confirmed that Cur-PCL spheroids had a significantly lower level of mitochondrial ROS compared to both control and PCL groups, consistent with the reported role of Cur in scavenging MitoROS and thus preserving mitochondrial function.

**Figure 5:**
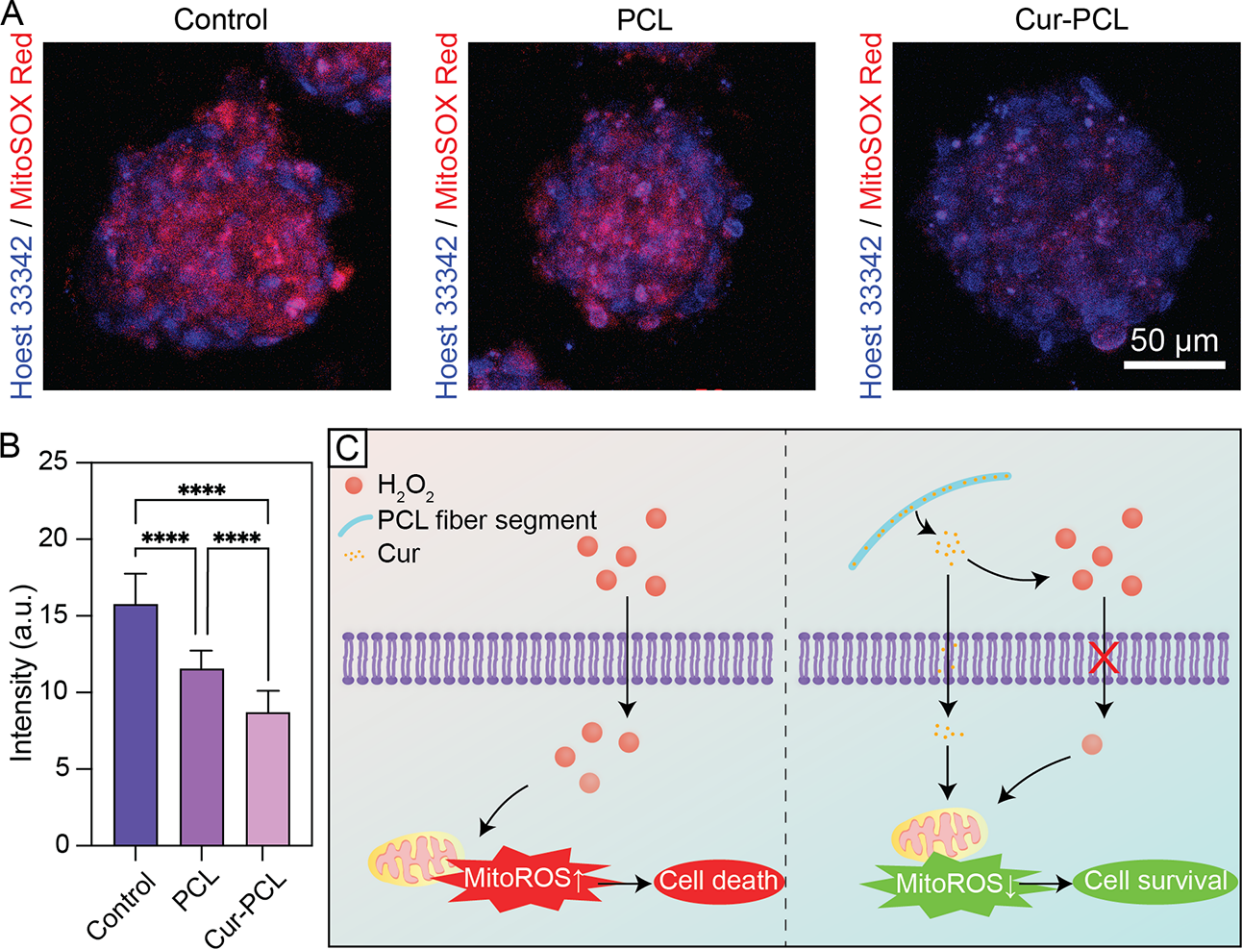
(A) Immunofluorescent staining of Hoechst 33342 (blue) and MitoSOX Red (red) in spheroids in the cases of control (MSCs only), PCL fiber segments, and Cur-PCL fiber segments, respectively, under oxidative stress. (B) Quantification of MitoSOX Red fluorescence intensity. (C) Schematic showing the presence of Cur-PCL fiber segments for cell protection against excessive ROS-induced damage.

The results are further summarized in Figure 5C, which illustrates the proposed mechanism. For the untreated spheroids (control group), the exposure to H_2_O_2_ resulted in excessive MitoROS accumulation, ultimately leading to cell death. In contrast, the Cur-PCL fiber segments released Cur, which could intercept and neutralize ROS, preventing their buildup inside mitochondria. Altogether, the Cur-PCL fiber segments could resist oxidative stress and regulate mitochondrial homeostasis, thereby supporting cell survival. The result highlights the potential of Cur-PCL fiber-based spheroids as multifunctional therapeutic platforms that combine structural support and antioxidant protection for regenerative medicine and tissue repair in ROS-challenged environments, offering distinct advantages over conventional nano- and microparticle-based systems.

Collectively, this fiber-integrated spheroid strategy holds promise for regenerative medicine applications involving cell delivery into oxidative-stress-rich environments, such as ischemic, inflamed, or injured tissues. The localized Cur release provides cytoprotection during the early post-transplantation window. Furthermore, the modular fiber-based design could be adapted to load alternative therapeutic agents, enabling broad applicability in cell-based therapies where enhanced survival and functionality of spheroids are critical.

## 4 Conclusion

In this study, we developed a fiber-integrated 3D spheroid platform by embedding Cur-loaded electrospun PCL fiber segments directly within stem cell spheroids. This coassembly approach enabled a uniform distribution of therapeutic fibers, sustained Cur release, and close cell-fiber interactions without disrupting spheroid integrity. The resulting constructs effectively mitigated oxidative stress, as demonstrated by reduced mitochondrial ROS accumulation and enhanced cell viability and proliferation under H_2_O_2_ challenge. By combining structural support with localized antioxidant delivery, this platform addresses key limitations of conventional strategies that rely on nanoparticle uptake or external scaffolds, which often suffer from poor integration, inconsistent drug release, or limited cellular interaction. The system offers an injectable solution to enhance the resilience and therapeutic performance of cell-based therapies in oxidative and inflammatory environments. Future work may explore its application in disease models and its adaptability to deliver other bioactive agents for specific tissue regeneration.

## Supplementary Material



## Notes

SEM image of PCL fiber; histograms of fiber segment length and diameter distribution; additional fields of view of Cur-PCL fiber segments; calibration curves and UV–vis spectra of Cur-PCL fiber segments dissolved in chloroform; encapsulation efficiency calculation; schematic diagram of the free radical scavenging reactions between DPPH· or ABTS+· and Cur-PCL fiber segments; fluorescent micrograph of the rhodamine-B labeled Cur-PCL fiber segments (PDF)
