# Supplementary material for: Short Segments of Electrospun Nanofibers Loaded with Curcumin Can Protect the Cells in Spheroids against Oxidative Stress
Source: ACS Appl Nano Mater. 2026 Feb 24;9(9):4346–55. doi: 10.1021/acsanm.6c00093 (PMC12973261; doi:10.1021/acsanm.6c00093)
Supplement: Supplementary file 1 [file an6c00093_si_001.pdf]

# Supporting Information

# Short Segments of Electrospun Nanofibers Loaded with Curcumin Can Protect the Cells in Spheroids against Oxidative Stress

Yuxuan Meng,[†, #] Min Hao,[‡, ǁ, #] and Younan Xia[†, ‡, ǁ, ¶, *]

[†]School of Chemistry and Biochemistry, Georgia Institute of Technology, Atlanta, Georgia 30332, United States

[‡]The Wallace H. Coulter Department of Biomedical Engineering, Georgia Institute of Technology and Emory University, Atlanta, Georgia 30332, United States

[ǁ]Department of Materials Science and Engineering, Johns Hopkins University, Baltimore, Maryland 21218, United States

[¶]Department of Biomedical Engineering, Johns Hopkins University, Baltimore, Maryland 21218, United States

[#]These authors contributed equally.

*Corresponding authors. E-mails: yxia70@jh.edu

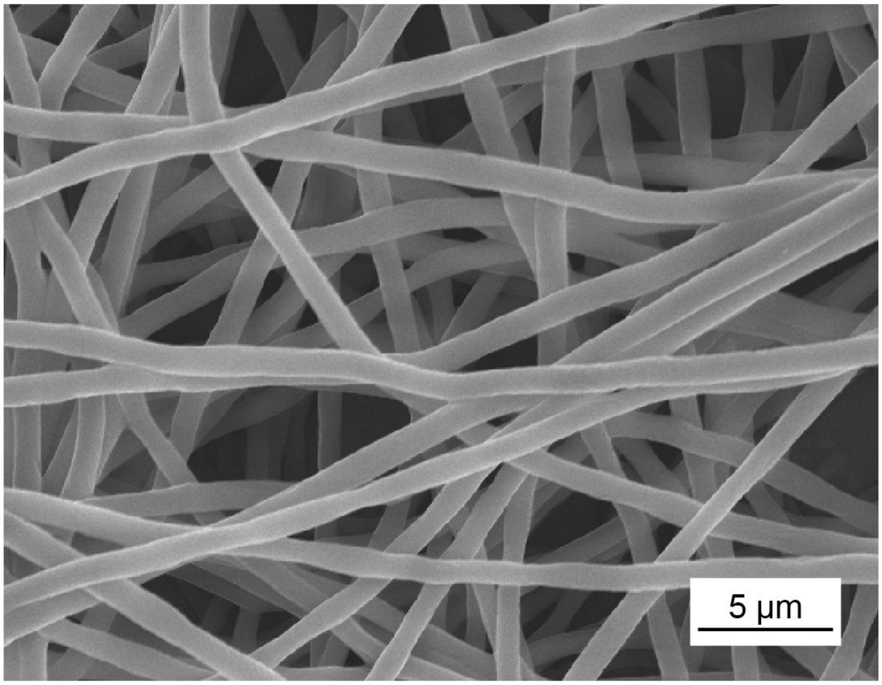


**Figure S1.** Scanning electron image of electrospun polycaprolactone (PCL) fibers.

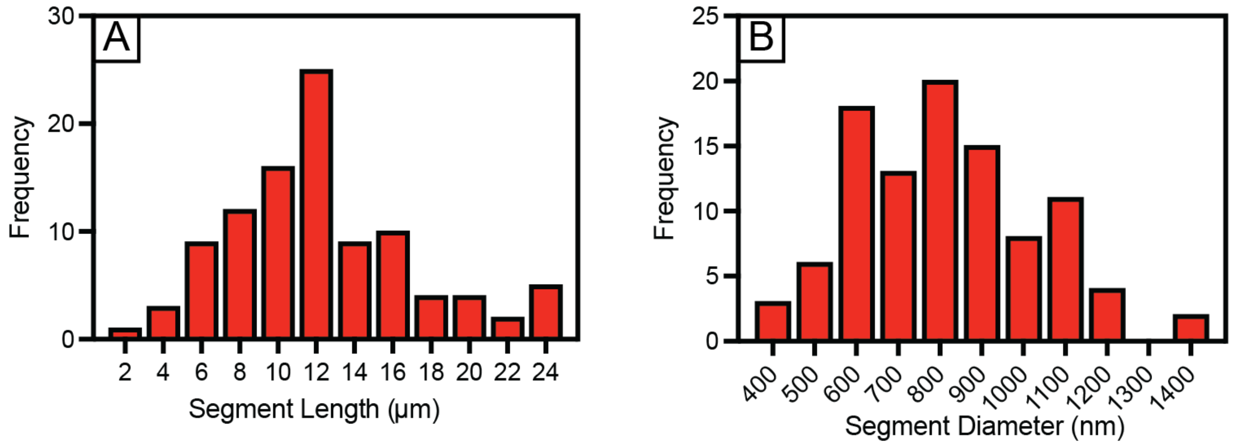


**Figure S2.** (A) Segment length distribution and (B) segment diameter distribution for homogenized Cur-PCL fiber segments, quantified from SEM images using ImageJ (n = 100 measurements). Data are reported as mean ± SD.

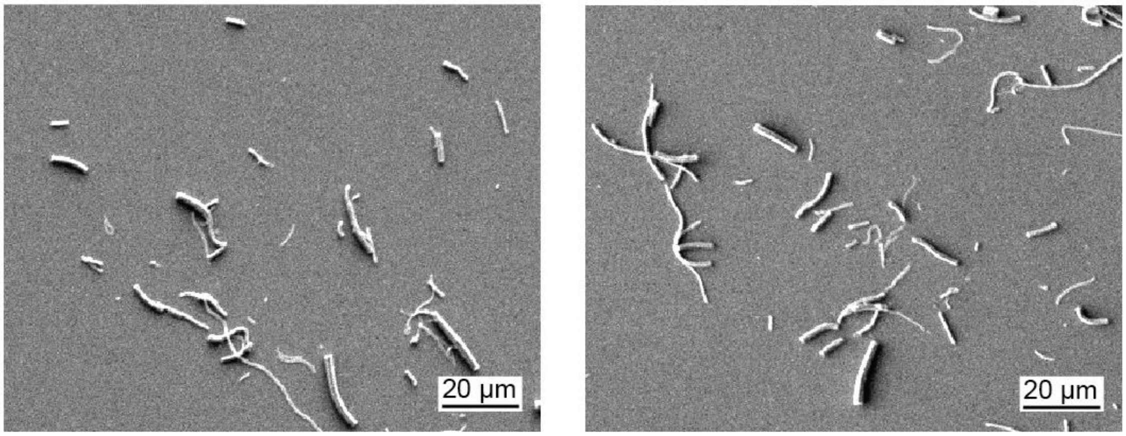


**Figure S3.** Representative SEM images showing additional fields of view of homogenized Cur-PCL fiber segments at different locations on the sample.

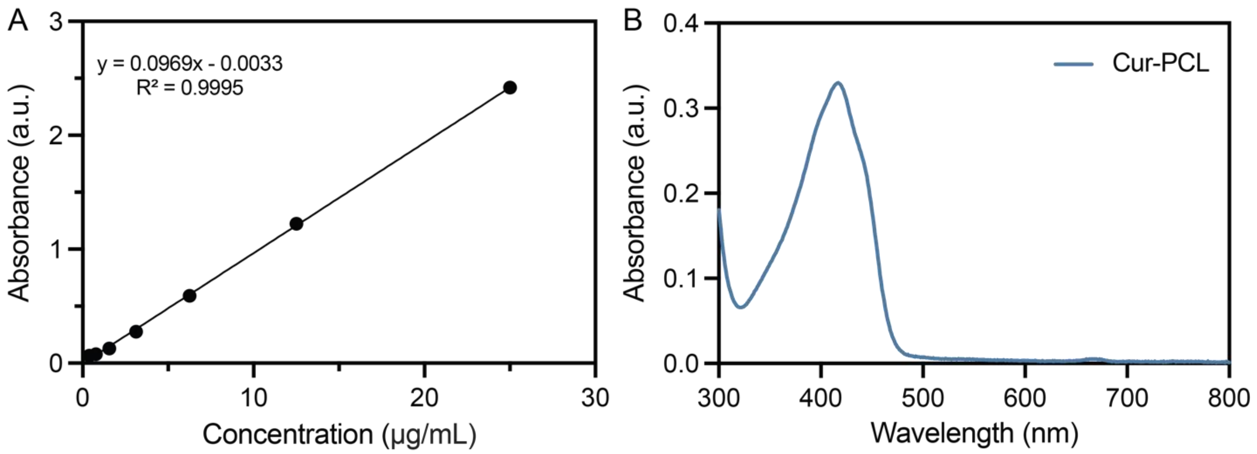


**Figure S4.** (A) Calibration curves used to calculate the encapsulation efficiency of curcumin (Cur). (B) UV-vis spectrum of Cur-PCL fiber segments dissolved in chloroform at 1:400 dilution.

$$Encapsulation\ efficiency\ (EE)\ (\%) = \frac{the\ amount\ of\ compound\ measured}{the\ theorical\ amount\ of\ compound} \times 100$$

$$EE\ (\%) = 58.55 \pm 7.59\ \%$$

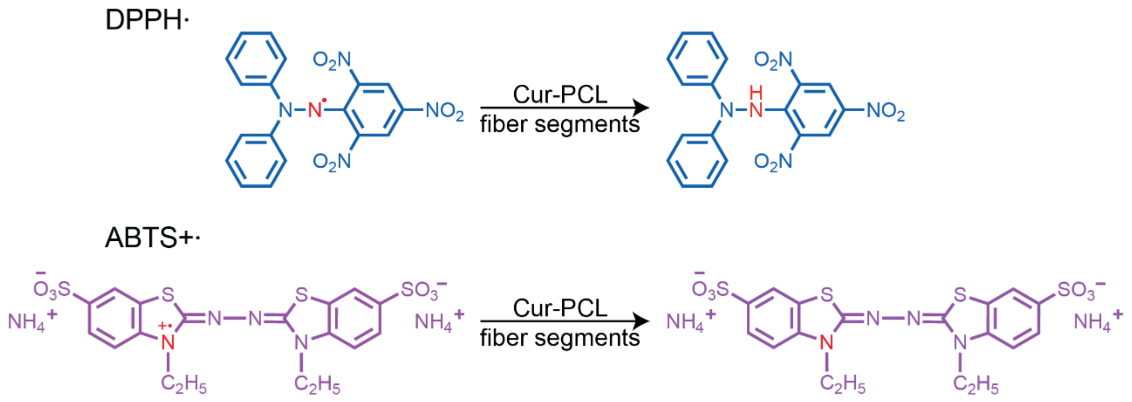


**Figure S5.** Schematic diagram of the free radical scavenging reactions between ,1-diphenyl-2-picrylhydrazyl free radical (DPPH·) or ethylbenzthiazoline-6-sulphonate (ABTS+·) and Cur-PCL fiber segments, which were used to assess the antioxidant activity.

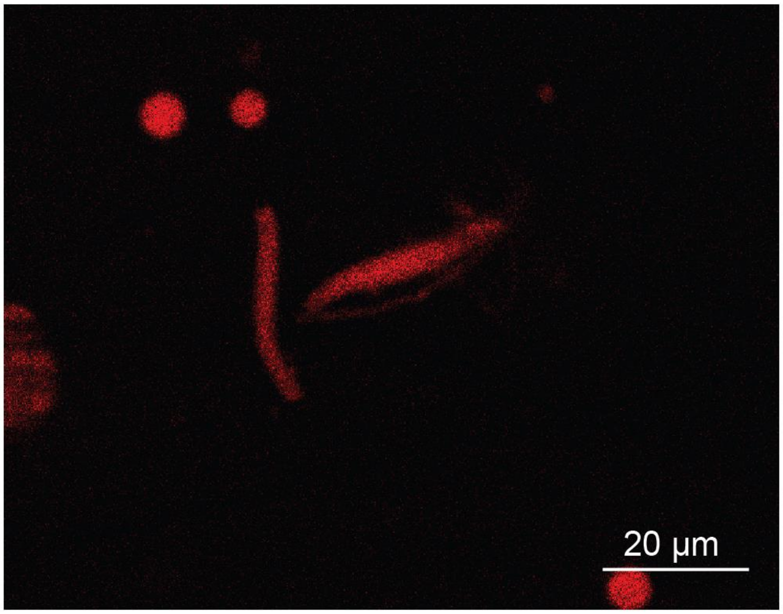


**Figure S6.** Fluorescent micrograph of the rhodamine-B labeled Cur-PCL fiber segments.
